# Supplementary figures and images for: HES1 is a novel downstream modifier of the SHH-GLI3 Axis in the development of preaxial polydactyly
Source: PLoS Genet. 2021 Dec 20;17(12):e1009982. doi: 10.1371/journal.pgen.1009982 (PMC8726490; doi:10.1371/journal.pgen.1009982)

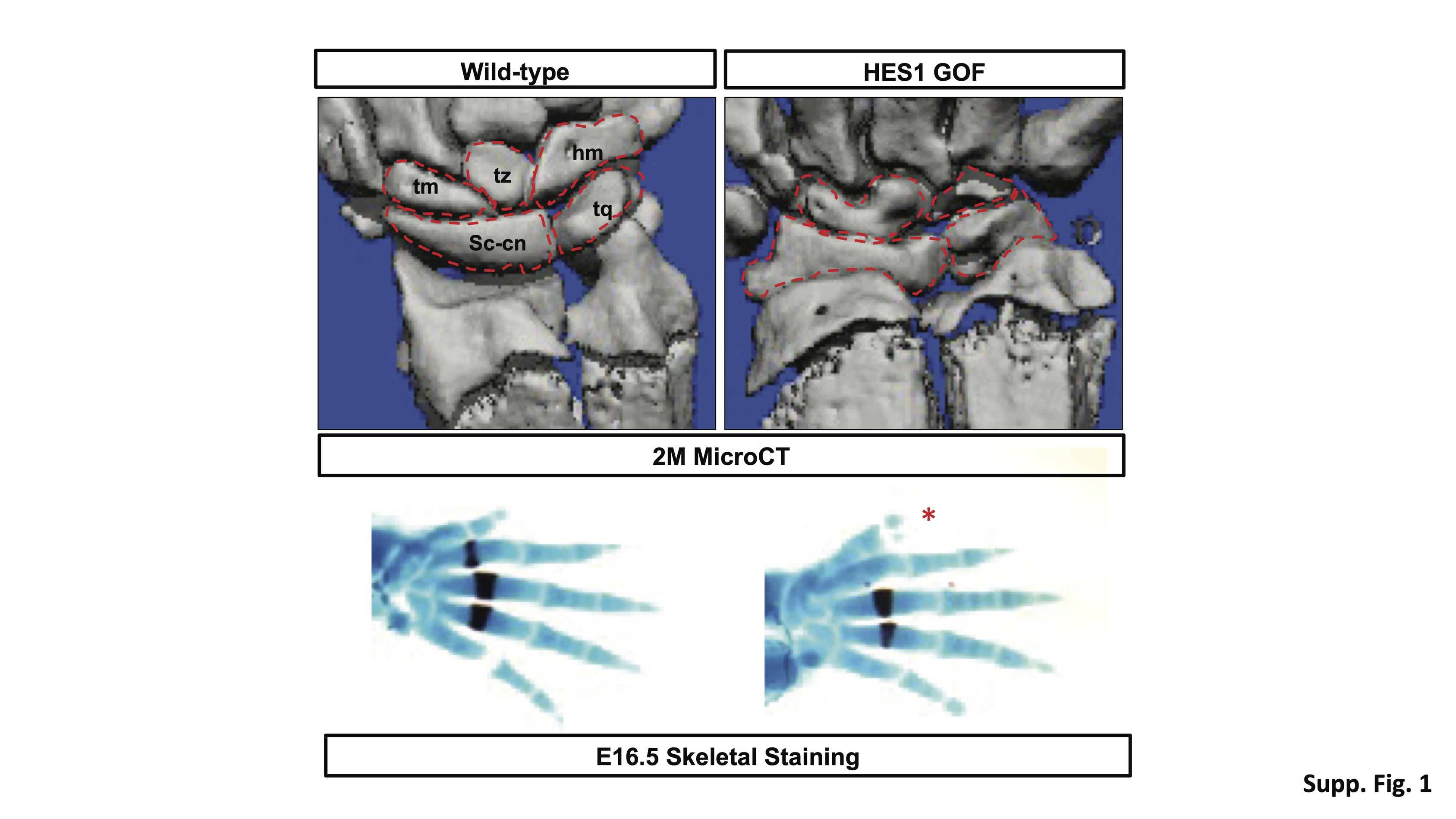

Supplement: S1 Fig — (A) MicroCT images of the carpal bones from WT and HES1 GOF mice at 2-month of age. hm, hamate; sc-cn, scaphoid-centrale; tq, triquetral; tm, trapezium; tz, trapezoid. Red dashed lines indicate location of carpal joints. (B) Alcian Blue/Alizarin Red staining of WT and Prx1Cre;R26-Hes1f/f (Hes1 GOF) mutant E16.5 forelimbs. Red asterisk indicates the syndactylous extra digit. (TIF) [file pgen.1009982.s001.tif]

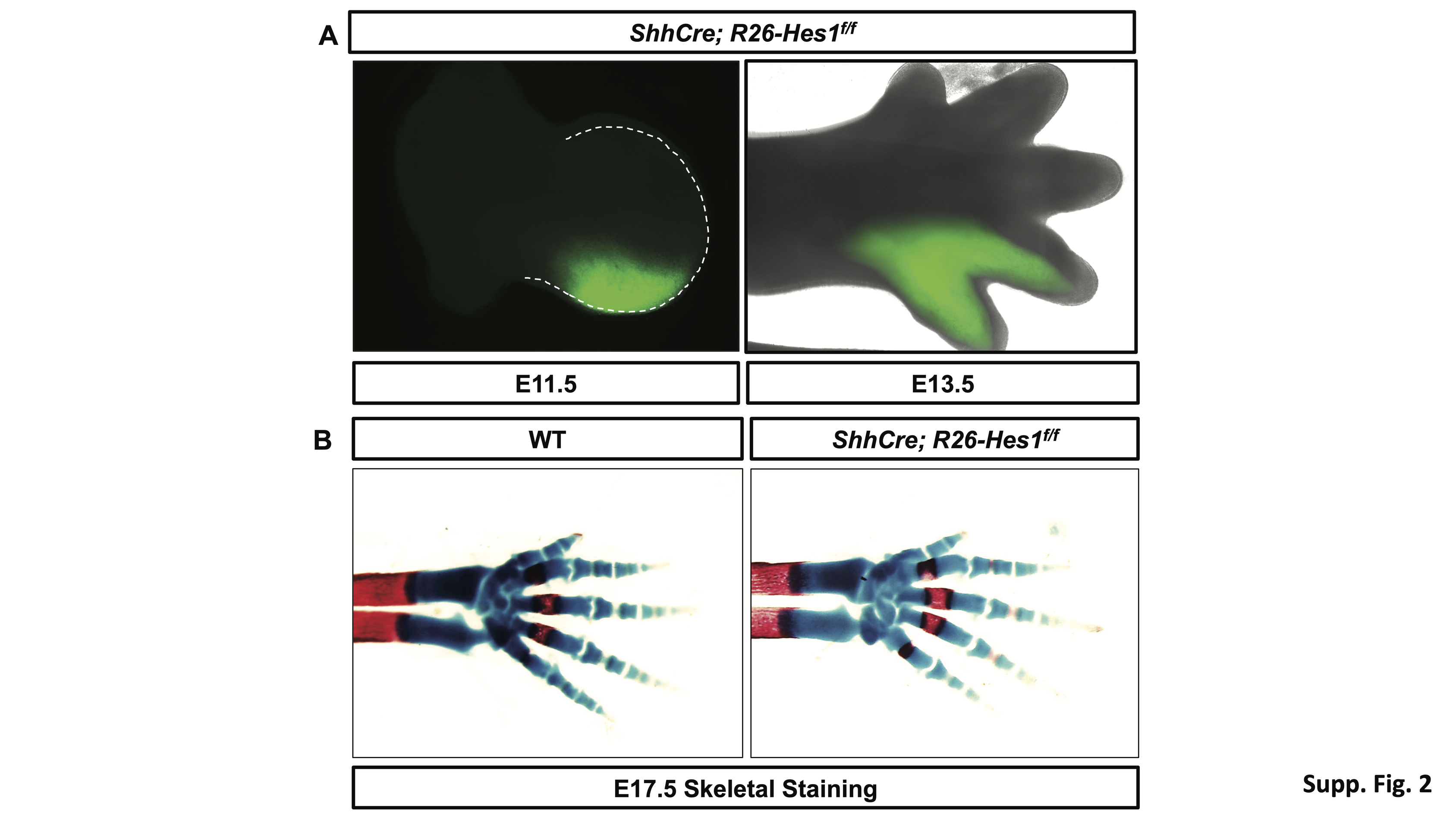

Supplement: S2 Fig — (A) GFP fluorescence from a ShhCre; R26-Hes1 f/f limb bud at E11.5 and forelimb at E13.5 (N = 6). R26-Hes1 floxed allele contains and IRES-GFP labeling ShhCre expressing cells and their descendants. (B) Alcian Blue/Alizarin Red staining of WT and ShhCre; R26-Hes1f/f mutant forelimbs at E17.5 (N = 12). (TIF) [file pgen.1009982.s002.tif]

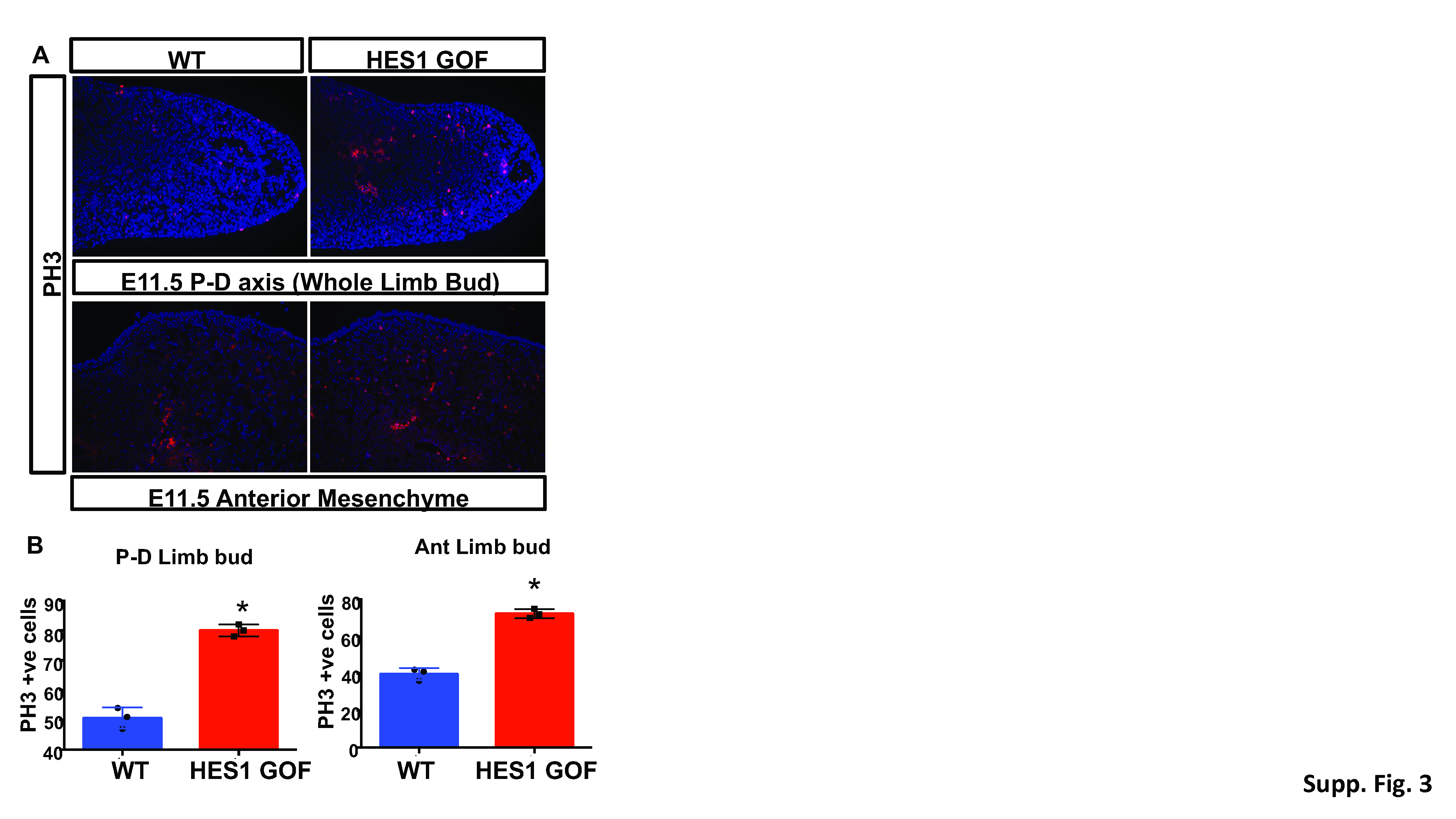

Supplement: S3 Fig — (A) Immunoflourescence for PH3 along the proximal-distal (P-D) axis and anterior mesenchyme of E11.5 HES1 GOF and WT limb buds (B) Quantification of PH3 positive cells along the P-D axis and anterior mesenchyme (N = 3). Asterisks indicate significance with a p-value < 0.05 (Student’s t-test). (TIF) [file pgen.1009982.s003.tif]

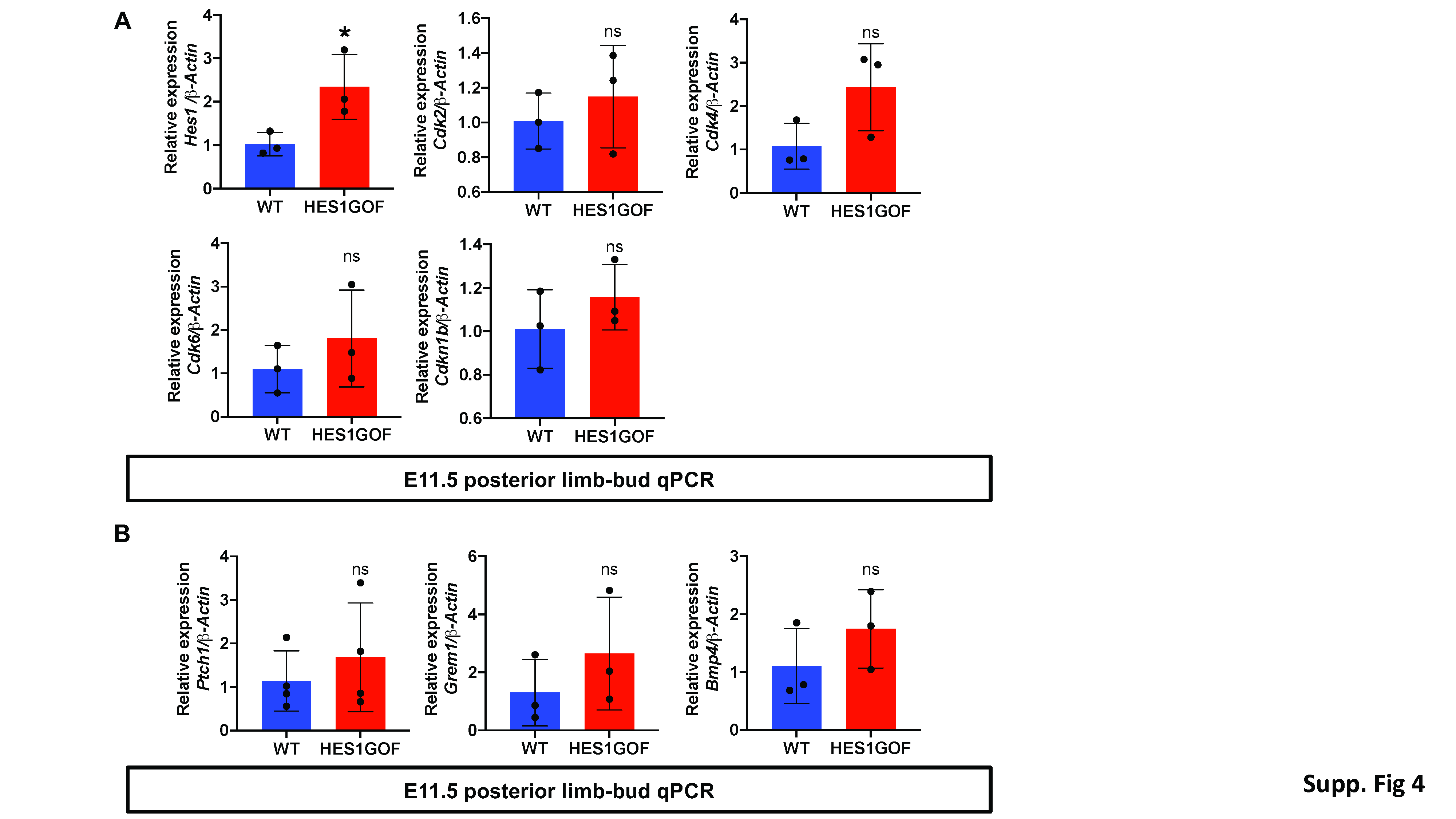

Supplement: S4 Fig — (A) qPCR for Hes1, Cdk2, Cdk4, Cdk6, and Cdkn1b on RNA isolated from WT and HES1 GOF E11.5 posterior halves of limb buds (N = 3). (B) qPCR for Ptch1, Grem1, and Bmp4 on RNA isolated from WT and HES1 GOF E11.5 posterior halves of limb buds (N≥3). Asterisks indicate significance with a p-value < 0.05 (Student’s t-test). (TIF) [file pgen.1009982.s004.tif]

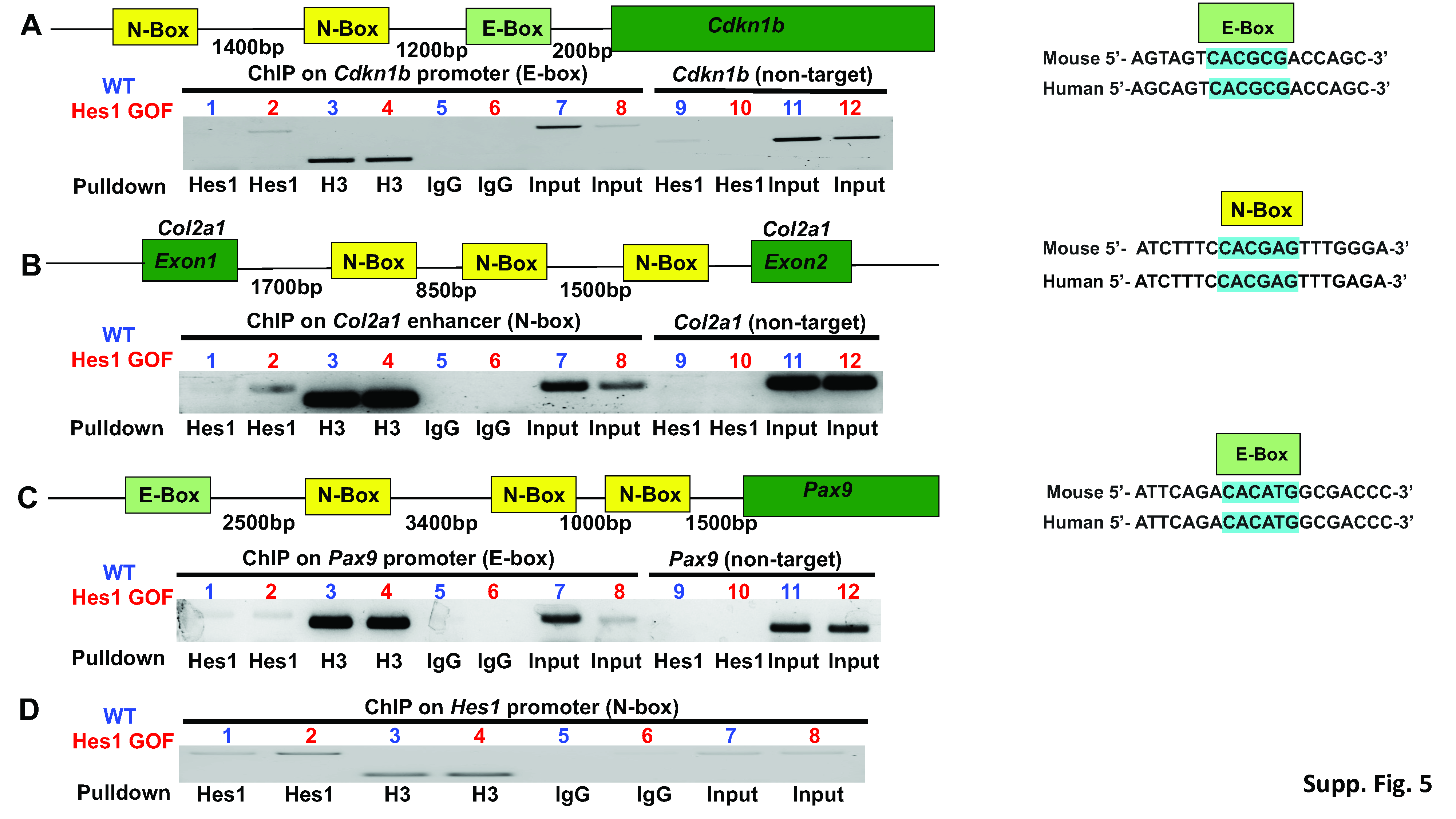

Supplement: S5 Fig — (A) Schematic of potential HES1 binding sites within the Cdkn1b promoter and a schematic of conserved E-Box (HES1 binding site) within human and mouse Cdkn1b promoters. (B) Schematic of potential HES1 binding sites within the Col2a1 enhancer region located between exons 1 and 2 and a schematic of the conserved N-Box within this region. (C) Schematic of potential HES1 binding sites within the Pax9 promoter and a schematic of the conserved E-Box between mouse and human. Approximate distances in base pairs (bp) upstream of transcriptional start sites or downstream from exons are indicated for each potential HES1 binding site. (A-D) Representative images of ChIP PCR and controls for HES1 binding of Cdkn1b, Col2a1, Pax9, and Hes1 promoters/enhancers (N = 3). N-box/E-box amplification of WT (Lane 1) and HES1 GOF (Lane 2) chromatin pulled down with anti-HES1. Amplification of WT (Lane 3) and HES1 GOF (Lane 4) chromatin pulled down with anti-HistoneH3 using RPL30 primers (positive controls). N-box/E-box amplification of WT (Lane 5) and HES1 GOF (Lane 6) chromatin pulled down with anti-IgG (negative controls). N-box/E-box amplification of WT (Lane 7) and HES1 GOF (Lane 8) input chromatin (positive controls). Non-target amplification upstream of N-box/E-box from WT (Lane 9) and HES1 GOF (Lane 10) chromatin pulled down with anti-HES1. Non-target amplification upstream of N-box/E-box from WT (Lane 11) and HES1 GOF (Lane 12) input chromatin. Non-target amplifications were not performed for the Hes1 promoter control (D; no lanes 9–12). (TIF) [file pgen.1009982.s005.tif]

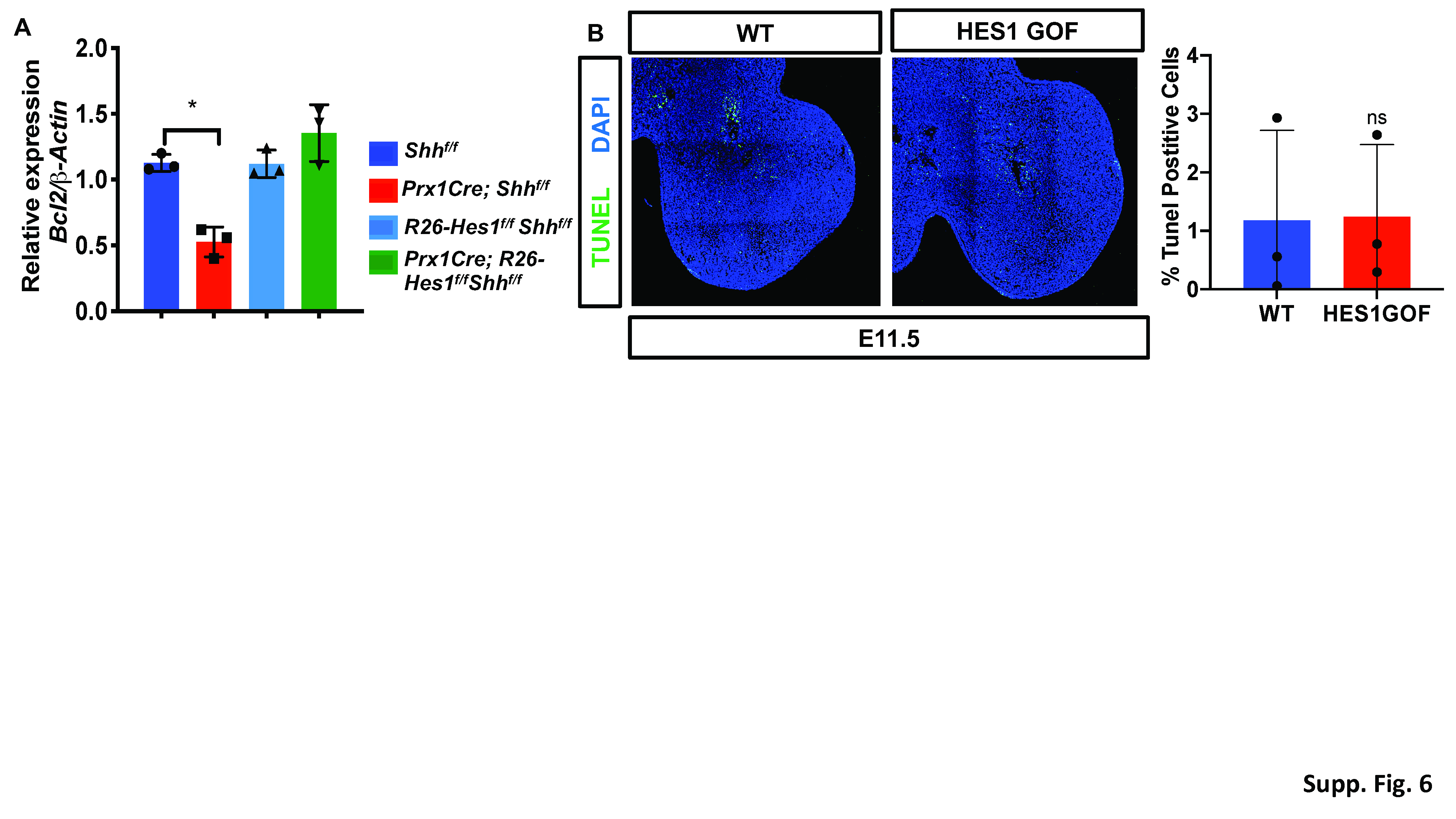

Supplement: S6 Fig — (A) qPCR for Bcl2 on RNA isolated from WT controls (Shhf/f)(R26-Hes1f/f; Shhf/f), SHH LOF (Prx1Cre; Shhf/f), and HES1 GOF/SHH LOF (Prx1Cre; R26-Hes1f/f; Shhf/f) E11.5 limb buds (N = 3). Asterisks indicate significance with a p-value < 0.05 (Student’s t-test). (B) TUNEL staining and quantification of WT and HES1 GOF limb bud sections at E11.5 (N = 3). (Student’s t-test). (TIF) [file pgen.1009982.s006.tif]

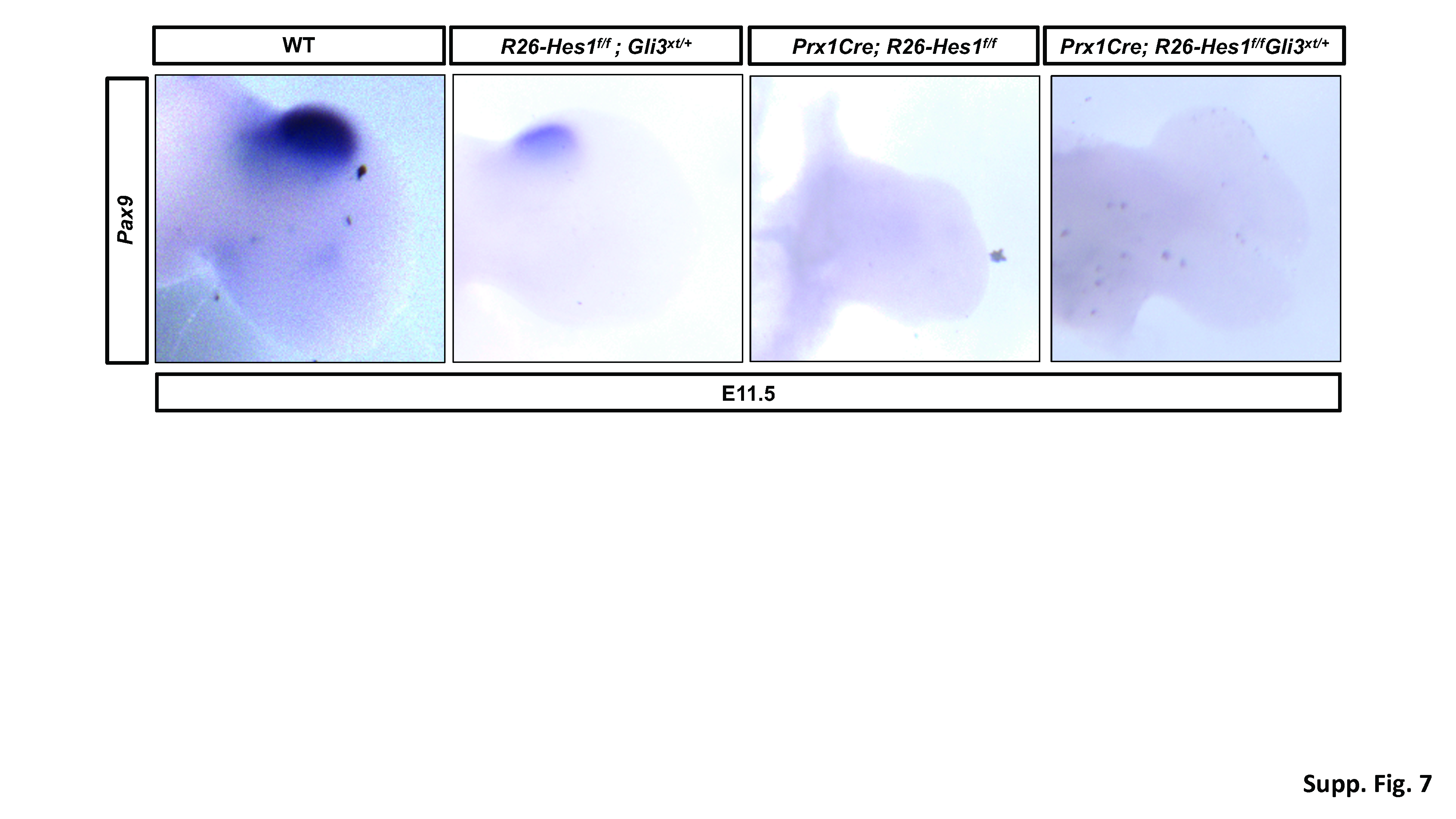

Supplement: S7 Fig — WISH for Pax9 on E11.5 WT, R26-Hes1f/f; Gli3xt/+ (GLI3 HET), Prx1Cre; R26-Hes1f/f (HES1 GOF), and Prx1Cre; R26-Hes1f/f; Gli3xt/+ (HES1 GOF/GLI3 HET) double mutant forelimbs. (N = 2). (TIF) [file pgen.1009982.s007.tif]

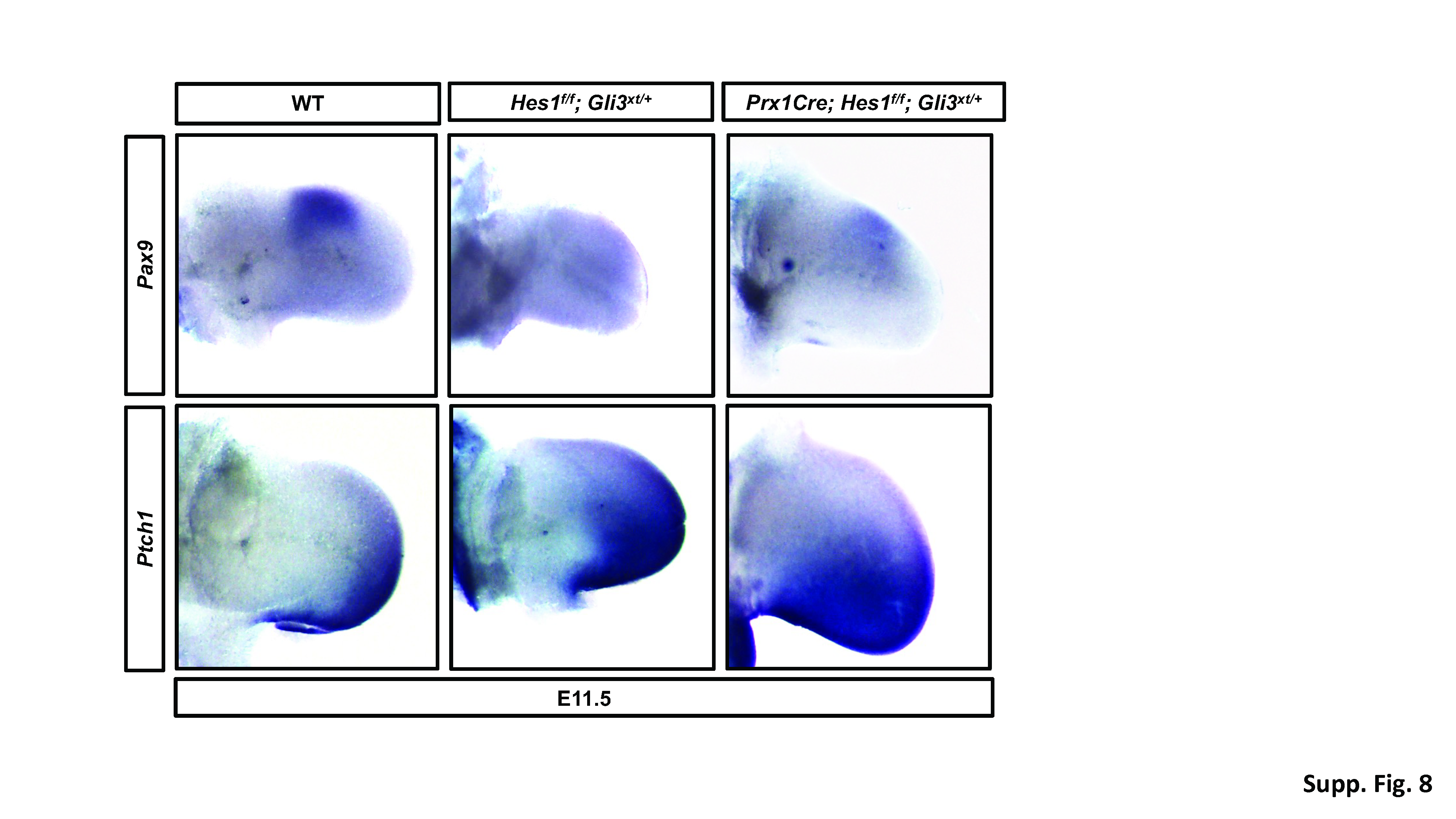

Supplement: S8 Fig — WISH for Pax9 and Ptch1 on E11.5 WT, Hes1 f/f; Gli3xt/+ (GLI3 HET), and Prx1Cre; Hes1f/f; Gli3xt/+ (HES1 LOF/GLI3 HET) double mutant forelimbs (N = 3). (TIF) [file pgen.1009982.s008.tif]

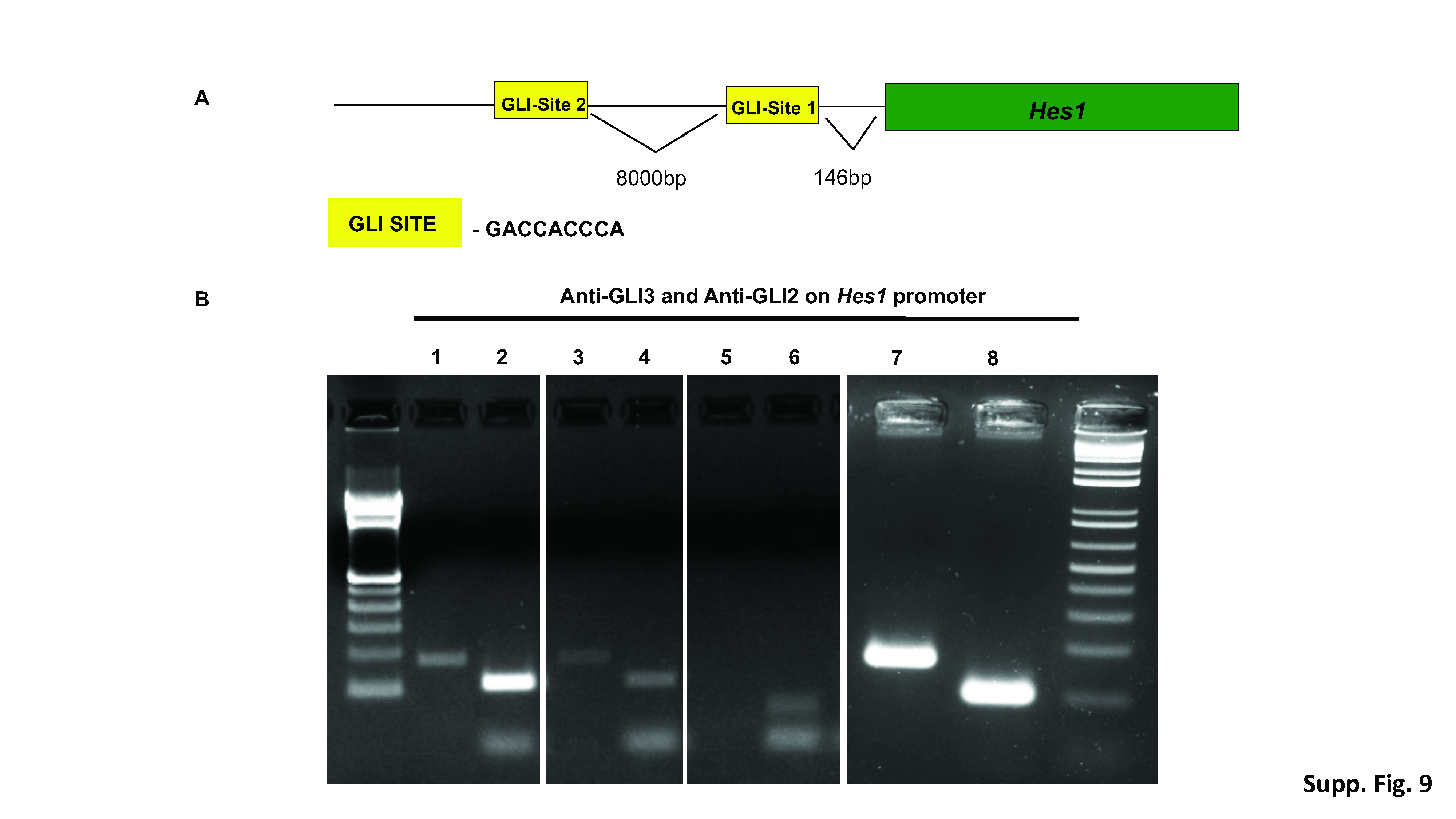

Supplement: S9 Fig — (A) Schematic of potential GLI binding sites within the Hes1 promoter. (B) ChIP and PCR amplification of chromatin containing GLI binding sites within Hes1 promoter (N = 3). Lane 1 = amplification of Site 1 using WT chromatin pulled down with anti-GLI3; Lane 2 = amplification of Site 2 using WT chromatin pulled down with anti-GLI3; Lane 3 = amplification of Site 1 using WT chromatin pulled down with anti-GLI2; Lane 4 = amplification of Site 2 using WT chromatin pulled down with anti-GLI2; Lane 5 = amplification of Site 1 using WT chromatin pulled down with anti-IgG (negative control); Lane 6 = amplification of Site 2 using WT chromatin pulled down with anti-IgG (negative control); Lane 7 = amplification of Site 1 using input control chromatin (positive control); Lane 8 = amplification of Site 2 using input control chromatin (positive control). (TIF) [file pgen.1009982.s009.tif]
