# Supplementary material for: HES1 is a novel downstream modifier of the SHH-GLI3 Axis in the development of preaxial polydactyly
Source: PLoS Genet. 2021 Dec 20;17(12):e1009982. doi: 10.1371/journal.pgen.1009982 (PMC8726490; doi:10.1371/journal.pgen.1009982)
Supplement: S1 Table — (DOCX) [file pgen.1009982.s010.docx]

**S1 Table. Real-time qPCR and ChIP primer sequences.**

| *Gene* | Forward Primer | Reverse Primer | Tm (°C) |
| --- | --- | --- | --- |
| *βactin* | AGATGTGGATCAGCAAGCAG | GCGCAAGTTAGGTTTTGTCA | 58 |
| *Hes1* | TACCCCAGCCAGTGTCAACA | TCCATGATAGGCTTTGATGACTTTC | 58 |
| *Hey1* | GAAACTTGAGTTCGGCGCTGTGTT | AGATCCCTGCTTCTCAAAGGCACT | 58 |
| *Pax9* | TGGATGCTGAGACGAAACTG | ACGCAGTGAATGGATTGGAG | 60 |
| *Grem1* | CAGTGTATGCGGTGCGAT | GCTCTCCTTCGTCTTCCTC | 58 |
| *Hand1* | CGCCCTTTAATCCTCTTCTCG | GCTGAACTCAAAAAGACGGATG | 60 |
| *Acan1* | CGTGTTTCCAAGGAAAAGGA | TGTGCTGATCAAAGTCCA | 58 |
| *Col2a1* | ACTGGTAAGTGGGGCAAGAC | CCACACCAAATTCCTGTTCA | 58 |
| *Sox9* | AGGAAGCTGGCAGACCAGTA | CGTTCTTCACCGACTTCCTC | 58 |
| *Alx4* | ACCCAGGTTGCTCTCTTTG | GCGCATCTCTAACTTGCAGA | 58 |
| *Hand2* | CTTCACTGCTTGAGCTCCCA | GAGGAGAAGAGGAAGAAAGAG | 58 |
| *Shh* | GCATTTAACTTGTCTTTGCACCT | GATCACAAGAAACTCCGAACGA | 58 |
| *Ptch1* | GCACAAATCTTCCAACTTCCAT | CTCAACTCATGATACAGACTCCAA | 58 |
| *Cdkn1b* | GCAGTGATGTATCTAATAAACAAGGA | GAGCAGACGCCCAAGAAG | 58 |
| *Ccnd1* | GCTTCAATCTGTTCCTGGCA | CAACAACTTCCTCTCCTGCTAC | 58 |
| *Cdk6* | GACTCCACGTCTGAACTTCCAC | AAGTCCTGCTCCAGTCCA | 58 |
| *Gli3* | GCTGCATGAAGACTCACCA | AGCAACACTACCTCAAAGCG | 58 |
| *Gli1* | GGAAGTCCTATTCACGCCTTGA | CAACCTTCTTGCTCACACATGTAA | 58 |
| *Sox5* | AGCCGCAATGCAGGTTTCT | TTGTGCTCTTGTCTGTGTGAAT | 58 |
| *Sox6* | GGTCATGTTTCCCACCCACAA | TTCAGAGGGGTCCAAATTCCT | 58 |
| *Pax9*  *ChIP*  *(E-box)* | GCGCTCTGTGCTCCTTTTG | CCAGGATCTTGCTGACGCA | 60 |
| *Cdkn1b*  *ChIP*  *(E-box)* | CGGCCGTTTGGCTAGTTTGTTTGT | AACCCAGCCGCTCTC CAAACCTTG | 58 |
| *Col2a1 ChIP*  *(N-box)* | ACACCCCTCCTCTCCATCTT | TCATGAATGGGGCTTTTCTC | 58 |
| *Pax9*  *ChIP*  *(non-target)* | TGGAAAACCGTGTTGGCAAT | GCAATCGGGCATCTTTGCAG | 58 |
| *Cdkn1b*  *ChIP*  *(non-target)* | AAGGCCCCAAAGGATAGGGA | GCCACGGCAGCTCTATTCA | 58 |
| *Col2a1*  *ChIP*  *(non-target)* | ATGGTTGTCGTCATCCCTTCTGG | CACCCGGTTGTATCTGGACT | 58 |
| *Hes1 ChIP*  *(N-box)* | TGGGAAGTTTCACACGAGCC | ATCTGCCATTTCACCCCGAG | 58 |
