# Supplementary material for: HES1 is a novel downstream modifier of the SHH-GLI3 Axis in the development of preaxial polydactyly
Source: PLoS Genet. 2021 Dec 20;17(12):e1009982. doi: 10.1371/journal.pgen.1009982 (PMC8726490; doi:10.1371/journal.pgen.1009982)
Supplement: S2 Table — (DOCX) [file pgen.1009982.s011.docx]

**S2 Table. Mouse Line Glossary**

| **Mouse** |  |
| --- | --- |
| *Prx1Cre; Shh ^f/f^* | Shh LOF (Loss-of-function) |
| *ShhCre; R26-Hes1^f/^* | HES1 GOF (Gain-of-function); utilizing ShhCre |
| *Prx1Cre; R26-Hes1^f/f^* | HES1 GOF ; utilizing Prx1Cre |
| *Prx1Cre;R26-Hes1^f/f^; Shh^f/f^* | SHH LOF/HES1 GOF |
| *Gli3^xt/+^* | GLI3 HET |
| *Prx1Cre;R26-Hes1^f/f^; Gli3^xt/+^* | GLI3 HET/HES1 GOF |
| *Prx1Cre; Hes1 ^f/f^* | HES1 LOF |
| *Prx1Cre;Hes1^f/f^;Gli3^xt/+^* | HES1 LOF/GLI3 HET |
